# Quality of Life Assessment and Clinical Implications for Women with Endometriosis Through Validated Tools: A Narrative Review

**DOI:** 10.3390/medicina61101729

**Published:** 2025-09-23

**Authors:** Andrei Manu, Elena Poenaru, Florentina Duica, Alexandra Irma Gabriela Bausic, Bogdan-Catalin Coroleuca, Ciprian-Andrei Coroleuca, Cristina Iacob, Ioana Rosca, Elvira Bratila

**Affiliations:** 1 Doctoral School, “Carol Davila” University of Medicine and Pharmacy, 020021 Bucharest, Romania; andrei.manu@drd.umfcd.ro (A.M.); cristina-maria.iacob@drd.umfcd.ro (C.I.); 2 Department of Obstetrics and Gynecology, “Carol Davila” University of Medicine and Pharmacy, 050474 Bucharest, Romania; alexandra.bausic@umfcd.ro (A.I.G.B.); cip_coroleuca@yahoo.com (C.-A.C.); elvirabarbulea@gmail.com (E.B.); 3 Faculty of Medicine, “Carol Davila” University of Medicine and Pharmacy, 050474 Bucharest, Romania; ioana.rosca@umfcd.ro; 4 Department of Obstetrics and Gynecology, “Prof. Dr. Panait Sârbu” Clinical Hospital of Obstetrics and Gynaecology, 060274 Bucharest, Romania; ccoroleuca@yahoo.com

**Keywords:** endometriosis, quality of life, fertility, personalized medicine, quality assessment tools

## Abstract

*Aim*: The aim of the study was to synthesize validated patient-reported outcome measures (PROMs) for assessing health-related quality of life (HRQoL) in women with endometriosis and to outline their clinical implications. *Methods*: We conducted a narrative review of English-language literature indexed in PubMed, Scopus, Web of Science, and Cochrane Library, covering the period 2014–2024, with earlier seminal studies included where relevant. We focused on validated PROMs for QoL but also considered standardized tools such as the Endometriosis Fertility Index (EFI), rASRM, and #Enzian classifications, given their role in clinical interpretation and counseling. *Findings*: Generic instruments (SF-36, WHOQOL-BREF, EQ-5D), disease-specific tools (EHP-30, EHP-5), and fertility-related questionnaires (FertiQoL, FPI) have demonstrated validity and responsiveness; however, these are inconsistently applied in practice. Knowledge gaps remain regarding routine implementation, timing, and frequency of assessment, and integration with clinical staging or fertility indices (e.g., EFI). Global frameworks such as the WERF EPHect platform facilitate standardized clinical and surgical data capture, though their use is primarily in research rather than routine care. *Conclusions*: We recommend combining a disease-specific PROM (EHP-30/EHP-5) with a generic instrument (SF-36 or EQ-5D) and adding FertiQoL when fertility is relevant. PROMs should be collected longitudinally (baseline, post-intervention, follow-up) and interpreted alongside clinical context, including pain phenotype, surgical staging (#Enzian/rASRM), and fertility goals. Embedding PROMs into multidisciplinary pathways enables shared decision-making, individualized treatment planning, and improved comparability of patient-centered outcomes.

## 1. Introduction

Endometriosis is a chronic, estrogen-dependent inflammatory disorder characterized by the presence of endometrial-like tissue outside the uterine cavity. It affects approximately 10% of women of reproductive age worldwide [1]. Clinical manifestations commonly include chronic pelvic pain, dysmenorrhea, dyspareunia, gastrointestinal symptoms, and infertility [1,2].

The condition has a substantial negative impact on health-related quality of life (HRQoL), affecting multiple domains such as physical functioning, emotional well-being, interpersonal relationships, and professional productivity [3,4]. In a cross-sectional study of women with deep infiltrating endometriosis (DIE), reduced scores were observed in both generic (SF-36) and disease-specific (EHP-30) quality of life (QoL) [5].

According to the World Health Organization, quality of life is defined as an individual’s perception of their position in life in the context of culture, value systems, goals, expectations, and concerns [6]. Women with endometriosis frequently experience delayed diagnosis, stigmatization of chronic pain, and uncertainty regarding fertility, all of which amplify psychological stress and reduce QoL [3,7,8]. 

Psychological comorbidities such as depression, anxiety, and social isolation are highly prevalent among women with endometriosis and further compound the disease burden [4,9]. Pharmacological and surgical treatments are effective for managing pain and improving fertility in selected cases, but they often fail to restore QoL to normal levels, particularly in emotional and social dimensions [2,5,10]. These psychological consequences exacerbate the overall disease burden and can persist even after physical symptoms are addressed.

To capture the full impact of the disease, several validated QoL instruments are now used in both clinical practice and research. The most commonly employed tools include the Endometriosis Health Profile-30 (EHP-30), the 36-Item Short Form Survey (SF-36), and the EuroQol-5D (EQ-5D), which facilitate longitudinal assessment of symptoms and support tailored clinical decision-making [5,11].

Therefore, our objective is to provide a narrative synthesis of validated HRQoL patient-reported outcome measures used in endometriosis—covering generic, disease-specific, and fertility-related instruments—while also briefly reviewing standardized tools, such as EFI, rASRM, and #Enzian, to situate QoL findings within the broader context of disease severity and fertility prediction.

## 2. Materials and Methods

This article was conducted as a narrative review. Our primary aim was to summarize validated patient-reported outcome measures (PROMs) used to assess health-related quality of life (HRQoL) in women with endometriosis and to discuss their clinical implications. To achieve this, we performed a literature search in MEDLINE/PubMed, Scopus, Web of Science, and the Cochrane Library. The search covered publications mainly from the last 10 years (2014–2024), with earlier seminal works included when describing the development or validation of instruments. Both PROMs for QoL and standardized tools (EFI, rASRM, #Enzian) were considered, as they provide complementary perspectives relevant for clinical interpretation of QoL outcomes. Search terms combined “endometriosis” with “quality of life”, “patient-reported outcomes”, “EHP-30”, “EHP-5”, “SF-36”, “EQ-5D”, “WHOQOL-BREF”, and “FertiQoL”. Articles were selected based on their relevance to QoL assessment and instrument validation, and full texts considered important for clinical and research applicability were reviewed. Because our goal was to provide a broad and integrative overview, we did not apply duplicate screening, formal risk-of-bias assessment, or PRISMA reporting.

## 3. Discussion

### 3.1. Endometriosis Impact on Quality of Life and Fertility

As can be seen from numerous publications, an estimated 10% of women of reproductive age are affected by this disease, and of women suffering from infertility, a percentage between 25–50% are diagnosed with endometriosis [1,2]. The disease is characterized histologically by ectopic endometrial-like glands and stroma, primarily involving the ovaries, uterosacral ligaments, peritoneum, and rectovaginal septum [3,4]. Clinically, patients present with a spectrum of symptoms, including dysmenorrhea, chronic pelvic pain (both cyclic and acyclic), dyspareunia, dyschezia, and subfertility or infertility [3,5,6].

The broad symptomatology and uncertain correlation with lesion burden contribute to challenges in clinical diagnosis and treatment planning. Although some women (estimated 20–25%) remain asymptomatic, the majority report varying degrees of pain and reproductive dysfunction [2,4]. Notably, pain severity frequently lacks correlation with rASRM staging, but shows strong association with lesion topography, especially in DIE affecting rectovaginal structures and bowel that leads to dyspareunia, dyschezia, and profound impairment of daily functioning [5,7].

The impact of endometriosis on health-related quality of life (HRQoL) is multidimensional. Physical domains are affected by pain, fatigue, and reduced mobility; emotional health is compromised by anxiety, depressive symptoms, and uncertainty regarding fertility; and social life is disrupted due to work absenteeism, impaired intimate relationships, and social withdrawal [3,6,8,12].

A large cross-sectional study by De Graaff et al. (*n* = 1418) reported that 60% of women with endometriosis experienced limitations in social activities and 70% in occupational tasks due to pain or fatigue, with significant reduction in SF-36 scores in physical functioning, role-physical, and vitality domains compared to controls [6]. A systematic review by Hansen et al. identified pain, infertility, diagnostic delays, and poor patient–physician communication as central contributors to psychological distress and dissatisfaction with care [9].

Infertility itself is a major source of emotional burden. A recent observational study comparing infertile women with and without endometriosis revealed significantly lower Fertility Quality of Life (FertiQoL) scores in the endometriosis group, particularly in emotional and mind–body domains (*p* < 0.01) [10]. Moreover, women with endometriosis report elevated levels of fertility-related stress, concern about time pressure for conception, and fear of irreversible damage to reproductive capacity [11].

Depression and anxiety are highly prevalent. This underscores the necessity of integrating psychological screening into routine gynecologic evaluations for women with suspected or confirmed endometriosis. A 2020 meta-analysis including over 9000 patients across 28 studies found a pooled prevalence of depressive symptoms in 37% and anxiety symptoms in 43% of women with endometriosis, with higher rates among those with persistent pain or infertility [13]. Another large Brazilian cohort showed that women with moderate-to-severe pelvic pain had an almost four-fold higher risk of major depression compared to controls (OR 3.8; 95% CI 1.2–12.8) [14].

The World Endometriosis Research Foundation’s Endometriosis Phenome and Biobanking Harmonization Project (WERF EPHect) represents the largest coordinated international effort to standardize data collection in endometriosis research [15,16]. EPHect provides patient questionnaires and surgical forms that improve comparability across studies and registries [15,16]. However, it is important to note that EPHect is not a quality-of-life instrument, and its uptake in daily clinical practice remains limited. Its main role is within research standardization, where it can serve as a framework for aligning validated PROMs (such as EHP-30, SF-36, or FertiQoL) with harmonized clinical datasets, thereby improving the comparability of outcomes across different settings [17].

### 3.2. Validated Quality of Life Instruments 

The assessment of health-related quality of life (HRQoL) in women with endometriosis has become essential for understanding the full burden of the disease and guiding clinical decision-making. Given the heterogeneous manifestations and the multidimensional impact of symptoms such as chronic pain, fatigue, and infertility, validated instruments are necessary to capture physical, emotional, and social consequences in a reliable and reproducible manner [1,2,3].

These instruments fall into two main categories: generic tools, which allow for comparisons across different diseases and populations, and disease-specific tools, which provide detailed insights into the particular experiences of women living with endometriosis [4]. Among the most widely used generic instruments are the Short Form-36 Health Survey (SF-36), the World Health Organization Quality of Life—BREF (WHOQOL-BREF), and the EuroQol 5-Dimension questionnaire (EQ-5D), while disease-specific assessment is typically performed using the Endometriosis Health Profile-30 (EHP-30) [2,5,6].

Each of these instruments has undergone validation in various languages and cultural contexts and has demonstrated good psychometric properties, including internal consistency, construct validity, and responsiveness to clinical change [3,7]. The selection of a QoL assessment tool should be tailored to the clinical or research objective. For comparative studies across conditions, generic tools are preferred, while disease-specific instruments like EHP-30 offer greater sensitivity in endometriosis-focused evaluations.

#### 3.2.1. The Endometriosis Health Profile-30 (EHP-30)

The EHP-30 is a disease-specific instrument developed by Jones et al. in 2001 to measure the impact of endometriosis on patients’ quality of life over the previous four weeks [7]. It includes a 30-item core questionnaire covering five domains: pain, control and powerlessness, emotional well-being, social support, and self-image. A modular section includes 23 additional items addressing work, sexual relationships, infertility, interactions with healthcare professionals, and treatment satisfaction. Each item is scored on a scale from 0 (best possible QoL) to 100 (worst possible QoL) [7].

Validation studies have confirmed the EHP-30’s high internal consistency (Cronbach’s α > 0.85) and test–retest reliability across various populations [9,10]. In a Turkish adaptation, Darici et al. (2023) demonstrated strong construct validity and responsiveness among women undergoing medical and surgical management of endometriosis [10]. Cross-cultural validations in multiple languages, including Polish, French, and Portuguese, have confirmed its generalizability and applicability [11,13,14].

In a study of 58 women with deep infiltrating endometriosis, Yela et al. (2020) reported high completion rates of the core questionnaire, but lower response rates for the modular items, particularly in domains concerning infertility and children, reflecting their optional nature and the sensitivity of these topics [9]. Notably, psychological and emotional domains consistently scored better than physical domains such as pain and fatigue, emphasizing the somatic burden of the disease on HRQoL.

Aubry et al. (2017) compared the EHP-5 (a shortened version of the EHP-30) with the EQ-5D in women treated for endometriosis and found that the EHP-5 was more sensitive to changes in pain and emotional functioning, suggesting the superiority of disease-specific instruments in capturing relevant therapeutic outcomes [18].

#### 3.2.2. The 36-Item Short Form Health Survey (SF-36)

The SF-36 is a generic, self-administered questionnaire widely used to assess health-related quality of life (HRQoL) across various clinical populations and interventions. It comprises 36 items grouped into eight domains: physical functioning, role limitations due to physical problems, bodily pain, general health perceptions, vitality, social functioning, role limitations due to emotional problems, and mental health. These are summarized into two component scores: Physical Component Summary (PCS) and Mental Component Summary (MCS), both scaled from 0 (worst) to 100 (best) [19].

In the context of endometriosis, the SF-36 has been extensively applied to quantify the physical and psychological consequences of the disease and evaluate treatment outcomes. Women with endometriosis, especially those with deep infiltrating endometriosis (DIE), consistently report significantly lower scores in the physical domains—particularly physical functioning, bodily pain, and role-physical—compared to healthy controls [20,21]. These impairments are strongly correlated with pain severity, lesion localization, and disease duration rather than surgical staging alone [21].

In a large multicenter validation study involving 450 patients with moderate-to-severe endometriosis-associated pain, Stull et al. demonstrated that the SF-36 had high internal consistency (Cronbach’s alpha > 0.80) and strong convergent validity with other pain and function measures. The tool was sensitive to change following hormonal and surgical therapies, with PCS improvements exceeding the minimal clinically important difference of 5 points in treatment responders [20].

Another cross-sectional study by Facchin et al. emphasized the disproportionate impact of bodily pain and vitality scores in women with chronic pelvic pain, even in those with prior interventions [22]. These findings highlight the persistent somatic burden of endometriosis despite conventional treatment [22]. Furthermore, a meta-analysis by Mira et al. identified the SF-36 as the most frequently used generic instrument across 34 studies involving over 6000 patients, and confirmed its responsiveness, particularly in physical domains, after medical or surgical therapy [23].

However, despite its widespread use and strong psychometric profile, the SF-36 lacks sensitivity in capturing endometriosis-specific dimensions such as dyspareunia, infertility distress, or dissatisfaction with care, which are better addressed by disease-specific instruments like the EHP-30. Thus, current best practice recommends using the SF-36 in conjunction with a targeted questionnaire to ensure a comprehensive QoL assessment in this patient population [24].

#### 3.2.3. The EuroQol-5D (EQ-5D) 

The EQ-5D is a generic, preference-based instrument used to measure health-related quality of life (HRQoL) across a wide range of diseases. It comprises five dimensions—mobility, self-care, usual activities, pain/discomfort, and anxiety/depression—each scored on three or five levels. Additionally, it includes a visual analog scale (EQ VAS), on which patients rate their perceived overall health on a scale from 0 (worst imaginable) to 100 (best imaginable) [18].

In the context of endometriosis, EQ-5D has been employed primarily in economic evaluations and broad clinical assessments due to its brevity and ability to generate utility scores for quality-adjusted life year (QALY) calculations. However, its applicability to the endometriosis population remains limited by the tool’s reduced sensitivity to condition-specific symptoms, particularly infertility-related distress and sexual dysfunction [25].

Despite this limitation, studies have demonstrated acceptable psychometric properties of EQ-5D in endometriosis populations. In a prospective study by Touboul et al., 159 women with deep infiltrating endometriosis (DIE) completed the EQ-5D and reported a mean utility index score of 0.77 (±0.14) and a mean EQ VAS score of 63.4 (±21.0). Lower scores were significantly associated with severe dyspareunia and rectal infiltration, supporting the instrument’s validity in assessing pain and emotional distress in advanced disease [26].

A comparative study by Aubry et al. evaluated EQ-5D and the disease-specific EHP-5 in 253 women treated for endometriosis. While both instruments detected post-treatment improvement, EHP-5 demonstrated superior sensitivity to changes in dysmenorrhea and emotional domains, highlighting the limited responsiveness of EQ-5D in domains not directly related to pain or general health perception [27].

Moreover, in a systematic review by Mira et al., the EQ-5D was identified as useful in health economic evaluations but was recommended to be used alongside disease-specific instruments to ensure a comprehensive assessment of HRQoL in women with endometriosis [28]. This approach aligns with current clinical practice guidelines, which emphasize the complementary use of both generic and condition-specific tools for a complete evaluation of the patient experience [29].

### 3.3. Fertility-Related Tools

Infertility represents a major physical and emotional burden for women with endometriosis, affecting up to 30–50% of patients [29]. A significant proportion experience anxiety and psychological distress related to the uncertainty of future conception [30]. In a comparative survey of 70 women, 71% of infertile participants with endometriosis reported significant anxiety regarding fertility, compared to only 40% of fertile controls [31].

To assess the emotional and social impact of infertility, several validated instruments have been developed. The Fertility Quality of Life (FertiQoL) questionnaire is the most widely used tool to evaluate health-related quality of life (HRQoL) in individuals with fertility problems. Developed by Boivin et al. in collaboration with the ESHRE Psychology Guideline Development Group, FertiQoL includes 34 items across four core domains (emotional, mind–body, relational, and social) and two optional treatment domains (treatment environment and tolerability). Psychometric testing demonstrated good internal consistency (Cronbach’s α = 0.88–0.93), and the tool has been validated in more than 20 languages [32,33].

Other instruments, such as the Fertility Problem Inventory (FPI) and the Fertility Problem Stress (FPS) scales, assess infertility-specific stress, including concerns related to social stigma, sexual life, and relationship strain. The FPI, originally developed by Newton et al., contains 46 items and has been extensively used in psychosocial fertility research [34,35].

A 2023 review confirmed the reliability of FertiQoL in capturing both general and treatment-related fertility stress, supporting its use in clinical and research settings involving patients with endometriosis-related infertility [34,35].

#### Endometriosis Fertility Index (EFI)

The Endometriosis Fertility Index (EFI) was developed by Adamson and Pasta in 2010 as a scoring system to predict non-assisted reproductive outcomes following surgical treatment for endometriosis [36,37]. The EFI combines historical variables (age, duration of infertility, prior pregnancy) and surgical findings (least function score, rAFS score) to generate a score between 0 and 10, with higher values indicating a greater likelihood of natural conception.

In their original study of 579 women, EFI scores ≥ 7 were associated with cumulative pregnancy rates over 50% within 36 months, while scores ≤ 3 predicted < 10% likelihood of conception [36,37]. These findings were validated externally in several cohorts. For example, Boujenah et al. confirmed the predictive value of the EFI in 180 patients undergoing conservative surgery, reporting significantly higher pregnancy rates in those with EFI ≥ 5 [38].

A meta-analysis by Tomassetti et al. concluded that EFI provides superior predictive accuracy for natural conception compared to the revised American Fertility Society (rAFS) classification alone [39]. Furthermore, the EFI can guide decision-making regarding the timing and appropriateness of assisted reproductive technologies (ART) such as in vitro fertilization (IVF) [40].

### 3.4. Endometriosis Staging Systems

#### 3.4.1. ASRM (rASRM) Classification

The Revised American Society for Reproductive Medicine (rASRM) classification, introduced in 1997, remains the most widely used surgical staging system for endometriosis [41]. It stratifies disease into four stages—minimal (I), mild (II), moderate (III), and severe (IV)—based on a point system that accounts for the size, depth, and location of endometriotic implants and the extent of pelvic adhesions involving the ovaries and fallopian tubes.

Despite its broad use, the rASRM classification presents notable limitations. It correlates poorly with symptom severity, particularly pain, and does not include deep infiltrating endometriosis (DIE) or extrapelvic lesions [42,43]. Symptoms like dysmenorrhea, dyspareunia, or infertility may be present even in Stage I disease, whereas some patients with Stage IV lesions may be asymptomatic, highlighting its limited prognostic value [42]. Moreover, the system fails to capture the heterogeneity of endometriosis phenotypes and lacks relevance in individualized treatment planning or fertility counseling.

#### 3.4.2. #ENZIAN Classification

Since 2020, the internationally accepted terminology is #Enzian, which replaced the earlier “Enzian” classification introduced in 2005 [44]. The #Enzian system has been endorsed by the international consensus working group as the standard for describing deep endometriosis, with applicability across surgery, MRI, and transvaginal ultrasound [44]. Compared to the original version, #Enzian offers improved reproducibility, a clearer correlation with symptoms, and greater utility for interdisciplinary communication [44]. Unlike rASRM, #ENZIAN evaluates the depth, size, and anatomical location of lesions, specifically in retroperitoneal compartments. It divides DIE into three main compartments:

- rectovaginal septum and vagina
- uterosacral ligaments and pelvic sidewall
- rectum and sigmoid colon

Each compartment is graded from 1 (<1 cm) to 3 (>3 cm), and additional extra-genital involvement is coded: FA (adenomyosis), FB (bladder), FI (intestinal lesions above sigmoid), FU (ureters), FO (other organs such as diaphragm, lung, abdominal wall) [45,46].

The revised #ENZIAN system also integrates a peritoneal score (P), ovarian involvement (O), and adhesions (T for tubo-ovarian unit) to provide a comprehensive surgical map of disease burden [47].

Studies confirm that the #ENZIAN score correlates significantly with clinical symptoms—particularly dyspareunia and dyschezia—and offers better reproducibility and interobserver agreement in DIE cases compared to rASRM [46,48]. Imaging-adapted #ENZIAN classifications have been validated for preoperative MRI and transvaginal ultrasound, improving interdisciplinary planning and facilitating non-invasive assessment [49,50].

Comparative tools for assessing quality of life are presented in Table 1.

## 4. Clinical Implications in Treatment Planning and Follow-Up

Assessment of quality of life (QoL) has emerged as a crucial component in the contemporary management of women diagnosed with endometriosis. The chronic and relapsing nature of this disease significantly affects patients’ physical, psychological, social, and sexual health, necessitating a patient-centered, multidisciplinary approach [51,52].

Laparoscopic excision is considered the gold standard for surgical management of endometriosis, aiming to reduce symptoms such as chronic pelvic pain, dysmenorrhea, dyspareunia, and infertility. Numerous prospective studies have confirmed significant postoperative improvements in pain and overall QoL using validated instruments such as the Endometriosis Health Profile-30 (EHP-30) and the Short-Form 36 Health Survey (SF-36) [42,53,54]. Abbott et al. demonstrated significant and sustained improvement in QoL up to five years postoperatively, particularly in physical, emotional, and social functioning domains, emphasizing the efficacy of surgical management in carefully selected patients [41].

However, despite effective surgical intervention, persistent symptoms—particularly chronic pelvic pain and deep dyspareunia—remain a challenging issue. A comprehensive prospective study by Vercellini et al. showed that approximately 25% of women continued to report significant deep dyspareunia following successful laparoscopic excision of deep infiltrating endometriosis (DIE), highlighting limitations of surgery alone and the necessity of integrating postoperative pelvic floor physiotherapy, psychological support, and sexual counseling in the standard postoperative care model [55].

Furthermore, chronic pelvic pain associated with endometriosis often persists independently of disease stage or the completeness of lesion excision. According to De Graaff et al., long-term QoL impairment was closely linked to ongoing pelvic pain rather than surgical outcomes alone, underlining the complex pathophysiological mechanisms underlying chronic pain conditions in endometriosis and the need for tailored multidisciplinary management strategies [56].

Validated QoL instruments such as EHP-30, SF-36, and the EuroQoL 5D (EQ-5D) have demonstrated significant predictive utility in clinical settings. Garry et al. demonstrated that preoperative QoL scores could effectively predict postoperative improvements and overall patient satisfaction, supporting their routine integration into preoperative counseling and postoperative monitoring protocols [57]. The integration of QoL metrics thus facilitates a comprehensive understanding of treatment outcomes beyond mere anatomical success, enhancing patient-centered care.

Socioeconomic factors further influence QoL outcomes in endometriosis patients, particularly following surgical intervention. Women from lower socioeconomic backgrounds frequently report reduced QoL, likely attributable to limited access to specialized multidisciplinary care, delayed diagnosis, and inadequate postoperative support. This emphasizes the necessity of personalized care plans addressing both clinical symptoms and individual patient circumstances, as highlighted by Fourquet et al. in their comprehensive study on the socioeconomic impact of endometriosis [58].

Given these considerations, clinicians are encouraged to implement a systematic and standardized approach to QoL assessment in both clinical practice and research. Routine use of QoL instruments provides critical insights into patient experiences, informs shared decision-making processes, and supports the adoption of a holistic, multidisciplinary care strategy, optimizing long-term outcomes for women affected by endometriosis [51,52,59,60].

## 5. Conclusions

The comprehensive assessment of health-related quality of life using rigorously validated instruments has become an indispensable element of contemporary clinical practice and research in endometriosis. Given the disease’s intricate and multifaceted nature, characterized by chronic pelvic pain, emotional distress, social limitations, and compromised fertility, the routine implementation of both generic (such as SF-36 and EQ-5D) and disease-specific tools (including EHP-30 and FertiQoL) is paramount for an accurate representation of patient experiences.

These validated questionnaires not only quantify the multidimensional burden of endometriosis but also facilitate personalized therapeutic strategies, enhancing clinical decision-making beyond mere anatomical or symptomatic improvement. Surgical and pharmacological interventions, despite their proven effectiveness in alleviating physical symptoms, often inadequately address the psychosocial and emotional domains of patients’ lives, thereby underscoring the importance of a holistic, multidisciplinary care model.

Moreover, the incorporation of specific scoring systems such as the Endometriosis Fertility Index (EFI), and detailed classifications like the revised ASRM and #ENZIAN, further refines individualized treatment planning, particularly in the context of fertility preservation and prediction of reproductive outcomes. The predictive value of these instruments significantly aids clinicians in counseling patients and setting realistic expectations, thus improving patient satisfaction and long-term adherence to treatment plans.

Ultimately, the integration of structured quality-of-life evaluations into routine gynecological practice empowers clinicians with critical insights, enabling more comprehensive patient management. This practice aligns closely with evolving paradigms in healthcare that emphasize patient-reported outcomes as central metrics for treatment success, thus advancing the standard of care provided to women affected by endometriosis.

## Figures and Tables

**Table 1:** Comparative tools for assessing quality of life.

Instrument	Type	Domains Assessed	No. of Items	Validation	Use in Endometriosis
EHP-30	Specific	Pain, emotions, support, self-perception	30 (+23)	Yes	Extended
EHP-5	Specific	General QoL domains	5	Yes	Rapid screening
SF-36	Generic	8 general health dimensions	36	Yes	Inter-population comparisons
EQ-5D	Generic	5 dimensions + VAS	5 (+VAS)	Yes	Cost-effectiveness analysis
WHOQOL-BREF	Generic	4 QoL domains	26	Yes	Overall utility
FertiQoL	Fertility	Emotions, body, relationships, social, treatment	34	Yes	Infertility subgroups

## Data Availability

The data are available from the corresponding author upon reasonable request.

## Abbreviations

The following abbreviations are used in this manuscript:

NCBI	National Center for Biotechnology Information
HRQoL	Health-Related Quality of Life
DIE	Deep Infiltrating Endometriosis
QoL	Quality of Life
PCS	Physical Component Summary
MCS	Mental Component Summary
